# Supplementary material for: Causal Relationship between Aging and Anorexia Nervosa: A White-Matter-Microstructure-Mediated Mendelian Randomization Analysis
Source: Biomedicines. 2024 Aug 16;12(8):1874. doi: 10.3390/biomedicines12081874 (PMC11351342; doi:10.3390/biomedicines12081874)
Supplement: Supplementary file 1 [file biomedicines-12-01874-s001.zip › Supplementary Figure/Figure S4/Singe SNP plot of MR analysis for TL to ubm-b-1759.pdf]

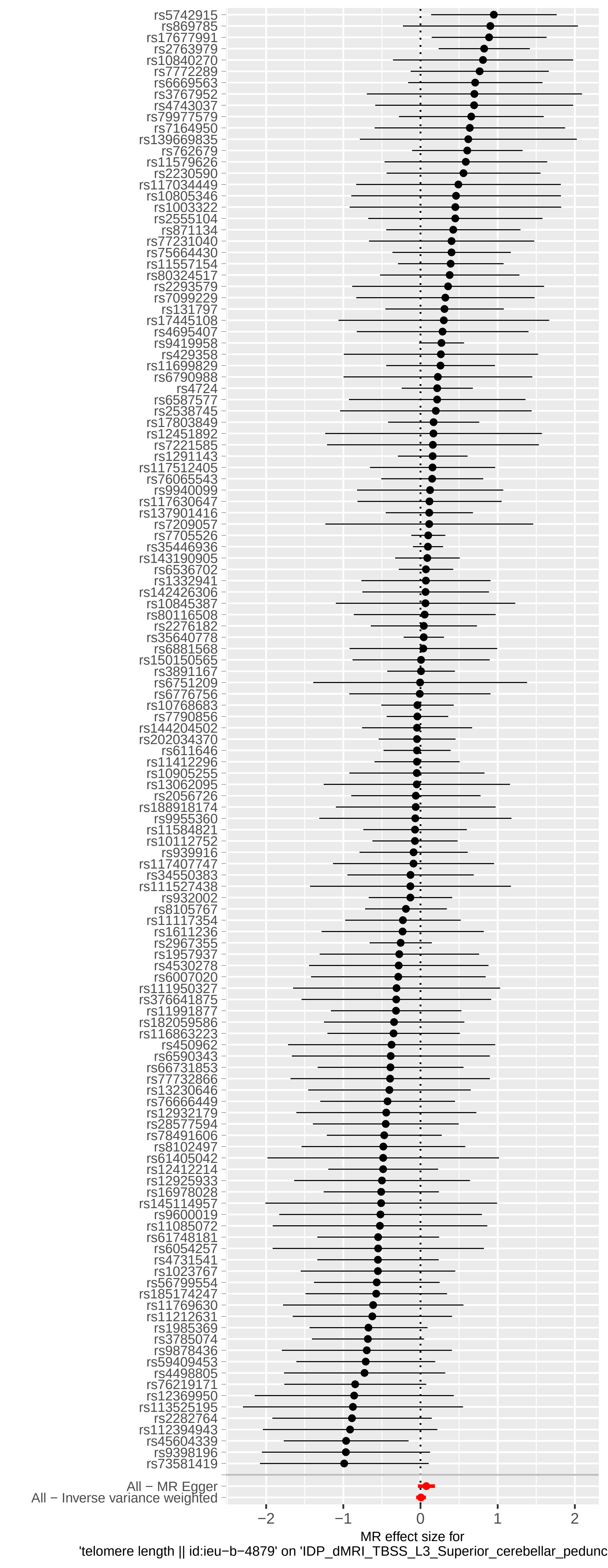
rs5742915
rs869785
rs17677991
rs2763979
rs10840270
rs7772289
rs6669563
rs3767952
rs4743037
rs79977579
rs7164950
rs139669835
rs762679
rs11579626
rs2230590
rs117034449
rs10805346
rs1003322
rs2555104
rs871134
rs77231040
rs75664430
rs11557154
rs80324517
rs2293579
rs7099229
rs131797
rs17445108
rs4695407
rs9419958
rs429358
rs11699829
rs6790988
rs4724
rs6587577
rs2538745
rs17803849
rs12451892
rs7221585
rs1291143
rs117512405
rs76065543
rs9940099
rs117630647
rs137901416
rs7209057
rs7705526
rs35446936
rs143190905
rs6536702
rs1332941
rs142426306
rs10845387
rs80116508
rs2276182
rs35640778
rs6881568
rs150150565
rs3891167
rs6751209
rs6776756
rs10768683
rs7790856
rs144204502
rs202034370
rs611646
rs11412296
rs10905255
rs13062095
rs2056726
rs188918174
rs9955360
rs11584821
rs10112752
rs939916
rs117407747
rs34550383
rs111527438
rs932002
rs8105767
rs11117354
rs1611236
rs2967355
rs1957937
rs4530278
rs6007020
rs111950327
rs376641875
rs11991877
rs182059586
rs116863223
rs450962
rs6590343
rs66731853
rs77732866
rs13230646
rs76666449
rs12932179
rs28577594
rs78491606
rs8102497
rs61405042
rs12412214
rs12925933
rs16978028
rs145114957
rs9600019
rs11085072
rs61748181
rs6054257
rs4731541
rs1023767
rs56799554
rs185174247
rs11769630
rs11212631
rs1985369
rs3785074
rs9878436
rs59409453
rs4498805
rs76219171
rs12369950
rs113525195
rs2282764
rs112394943
rs45604339
rs9398196
rs73581419
All − MR Egger
All − Inverse variance weighted
−2
−1
0
1
2
MR effect size for
'telomere length || id:ieu−b−4879' on 'IDP_dMRI_TBSS_L3_Superior_cerebellar_pedunc
